# Pseudo-Cushing Syndrome With an Atypically High Cortisol Burden and Clinical Improvement With Adrenal Enzyme Inhibitor

**DOI:** 10.1210/jcemcr/luad075

**Published:** 2023-07-01

**Authors:** Yeung-Ae Park, Frank Gao, Ie-Wen Sim, Chris Gilfillan

**Affiliations:** Department of Endocrinology and Diabetes, Eastern Health, Box Hill, Victoria 3128, Australia; Department of Diabetes and Endocrinology, Royal Melbourne Hospital, Parkville, Victoria 3050, Australia; Department of Endocrinology and Diabetes, Eastern Health, Box Hill, Victoria 3128, Australia; Department of Medicine, Austin Health, University of Melbourne, Heidelberg, Victoria 3084, Australia; Department of Endocrinology and Diabetes, Eastern Health, Box Hill, Victoria 3128, Australia; Melbourne Medical School, University of Melbourne, Parkville, Victoria 3010, Australia; Department of Medicine, School of Clinical Sciences, Monash University, Clayton, Victoria 3800, Australia; Department of Endocrinology and Diabetes, Eastern Health, Box Hill, Victoria 3128, Australia

**Keywords:** Cushing syndrome, hypercortisolism, pseudo-Cushing syndrome, malnutrition, nonneoplastic hypercortisolism

## Abstract

Distinguishing between Cushing syndrome (CS) and pseudo Cushing syndrome (PCS), also known as physiological hypercortisolism, can be difficult. PCS is caused by nonneoplastic overactivity of the hypothalamic-pituitary-adrenal axis and may be secondary to a range of conditions, including obesity, physical stress, malnutrition, and chronic alcoholism, and typically results in a lesser degree of hypercortisolism and fewer clinical features than CS. Management of PCS includes treatment of the underlying cause and reassessment of hypercortisolemia following improvement in the underlying etiology, as this may result in normalization of cortisol levels. The role of adrenal enzyme inhibitors in lowering cortisol levels in those with PCS is poorly understood. We report a case of a man presenting with weight loss who was found to have severe hypercortisolemia and elevated adrenocorticotropin (ACTH) complicated by infection, neuropsychiatric disturbance, and hypokalemia. Despite high cortisol levels, he was phenotypically not cushingoid, and the circadian rhythm of cortisol was preserved. Extensive investigations did not demonstrate a cause of symptoms or source of ACTH. Medical management with ketoconazole improved neuropsychiatric symptoms, and weight gain with nasogastric feeds resulted in the normalization of cortisol levels and resolution of symptoms following ketoconazole cessation.

## Introduction

Pseudo-Cushing syndrome (PCS), also known as nonneoplastic hypercortisolism, is due to physiological hyperactivation of the hypothalamic-pituitary-adrenal axis. Causes of PCS include depression, eating disorders, extreme physical stress, obesity, insulin resistance, and chronic alcoholism [1]. Differentiating between PCS and Cushing disease (CD) can be challenging because of their overlapping clinical and biochemical features. Furthermore, PCS and CD may not be distinguishable radiologically or with bilateral inferior petrosal sinus sampling (BIPSS) [2]. Although cortisol burden is typically lower in PCS compared with CD [3], there is a lack of data regarding the management of physiological hypercortisolism.

We present a case report of atypical PCS with severe hypercortisolism and dynamic test results mimicking CD, requiring treatment of PCS with an adrenal enzyme inhibitor as well as management of the underlying etiology of malnutrition.

## Case Presentation

A 56-year-old man presented with a 20-kg unintentional weight loss (from 60 to 40 kg) over 6 months and generalized weakness. He denied any infective symptoms or psychosocial stressors, and no cause for symptoms was apparent from his medical history. His only active health issue was gastroesophageal reflux disease, and he did not take any regular medications. Examination demonstrated cachexia and generalized weakness without a clear cause, as well as restricted affect and limited engagement. Serum potassium was low at 3.1 mmol/L (3.1 mEq/L) (reference range, 3.5-5.5 mmol/L [3.5-5.5 mEq/L]); otherwise, full blood count, renal function, thyroid function, and inflammatory markers were unremarkable.

## Diagnostic Assessment

**Figure 1.**
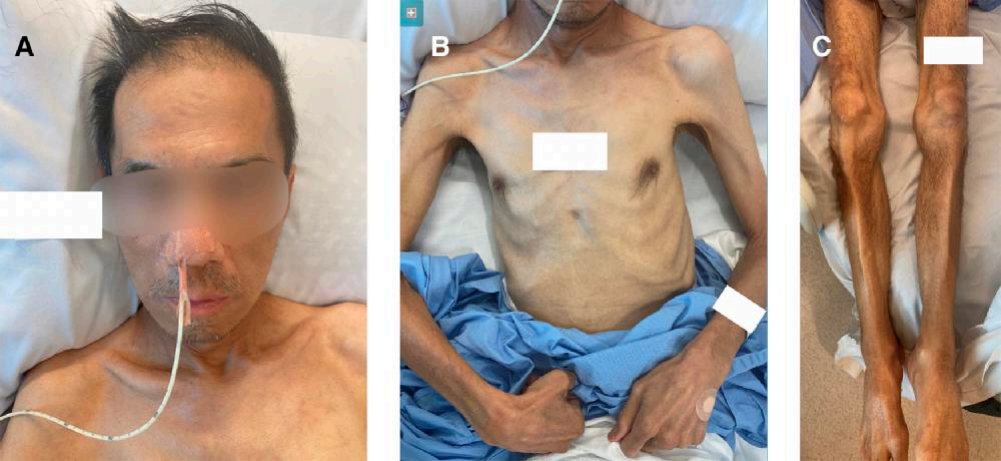
Photos of our patient: A, face; B, torso; and C, legs, demonstrating cachexia without features of Cushing syndrome.

Morning cortisol (performed to exclude adrenal insufficiency) was instead found to be markedly elevated at 4127 nmol/L (150 mcg/dL) (reference range, 110-550 nmol/L [4-20 mcg/dL]) with an elevated adrenocorticotropin (ACTH) level of 12 pmol/L (56 pg/mL) (reference range, < 10 pmol/L [<46 pg/mL]). Further screening tests were consistent with Cushing syndrome (CS) (Table 1). Repeat morning cortisol was 1645 nmol/L (59 mcg/dL) (reference range, 110-550 nmol/L [4-20 mcg/dL]) and midnight plasma cortisol was 727 nmol/L (26 mcg/dL) with midnight:morning plasma cortisol ratio 0.44. Despite the high cortisol burden, phenotypically he did not display any features of CS (Fig. 1).

**Table 1.** Comparison of cortisol burden and dynamic test results in pseudo-Cushing syndrome, Cushing disease, and our patient

	Pseudo-Cushing syndrome	Cushing disease	Our patient	Reference range for our patient's assays
24-h urinary free cortisol (4)	132 ± 119 nmol/24 h (47.8 ± 43.1 mcg/24 h)	1058 ± 1433 nmol/24 h (383.0 ± 518.7 mcg/24 h)	2017 nmol/24 h (730 mcg/24 h)	200-1000 nmol/24 h (73-363 mcg/24 h)
24 h urinary free cortisol (3)	698 ± 104 nmol/24 h (252.7 ± 37.6 mcg/24 h)	3371 ± 1027 nmol/24 h (1220.3 ± 371.8 mcg/24 h)	2017 nmol/24 h (730 mcg/24 h)	200-1000 nmol/24 h (73-363 mcg/24 h)
1-mg DST (3)	173 ± 52 nmol/L (6.3 ± 1.9 mcg/dL)	439 ± 39 nmol/L (15.9 ± 1.4 mcg/dL)	276 nmol/L (10.0 mcg/dL)	<50 nmol/L (<1.8 mcg/dL)
Midnight salivary cortisol (3)	7.7 ± 1.0 nmol/L (0.3 ± 0.04 mcg/dL)	35.0 ± 6.4 nmol/L (1.3 ± 0.2 mcg/dL)	105 nmol/L (3.8 mcg/dL)	<5 nmol (<0.2 mcg/dL)
Midnight serum cortisol (3) (sensitivity 98%, specificity 95%)	≤243 nmol/L (≤8.8 mcg/dL)	>243 nmol/L (>8.8 mcg/dL)	727 nmol/L (26.4 mcg/dL)	
Midnight:morning serum cortisol ratio (3) (sensitivity 87%, specificity 100%)	≤0.67	>0.67	0.44	
8 Am of day 2 serum cortisol level in 4-mg IV DST (4) (sensitivity 93.8%, specificity 83.3%)	≤130 nmol/L (≤4.7 mcg/dL)	>130 nmol/L (>4.7 mcg/dL)	717 nmol/L (26.0 mcg/dL)	
8 Am of day 2 ACTH level in 4-mg IV DST (4) (sensitivity 93.7%, specificity 88.9%)	≤4.31 pmol/L (≤19.6 pg/mL)	>4.31 pmol/L (>19.6 pg/mL)	5.94 pmol/L (27 pg/mL)	

Abbreviations: ACTH, adrenocorticotropin; DST, dexamethasone suppression test; IV, intravenous.

Extensive investigations including computed tomography and whole-body magnetic resonance imaging (MRI), 2 MRI pituitary scans, fluorodeoxyglucose, and Gallium-68 DOTATATE positron emission tomography scans, gastroscopy, colonoscopy, and tumor markers did not reveal a source of ACTH or other cause of his symptoms. Bronchoscopy was unremarkable with normal cytology. The remainder of the pituitary and adrenal panel were unremarkable.

Hypercortisolemia was complicated by an immunocompromised state with esophageal candidiasis, shingles, and a new cavitating *Streptococcus pneumoniae* lung infection. Secondary immunodeficiency screen including HIV was negative.

Initial psychiatry impression was of a major depressive disorder and possible underlying disordered eating, as collateral history revealed the patient had been a “picky eater.” He was commenced on escitalopram, which was subsequently ceased because of hyponatremia.

The patient continued to lose weight with a nadir body mass index (BMI) of 12.6 kg/m^2^ and nasogastric feeds were commenced, while oral potassium replacement up to 56 mmol/day was required to maintain normokalemia.

## Treatment

**Figure 2.**
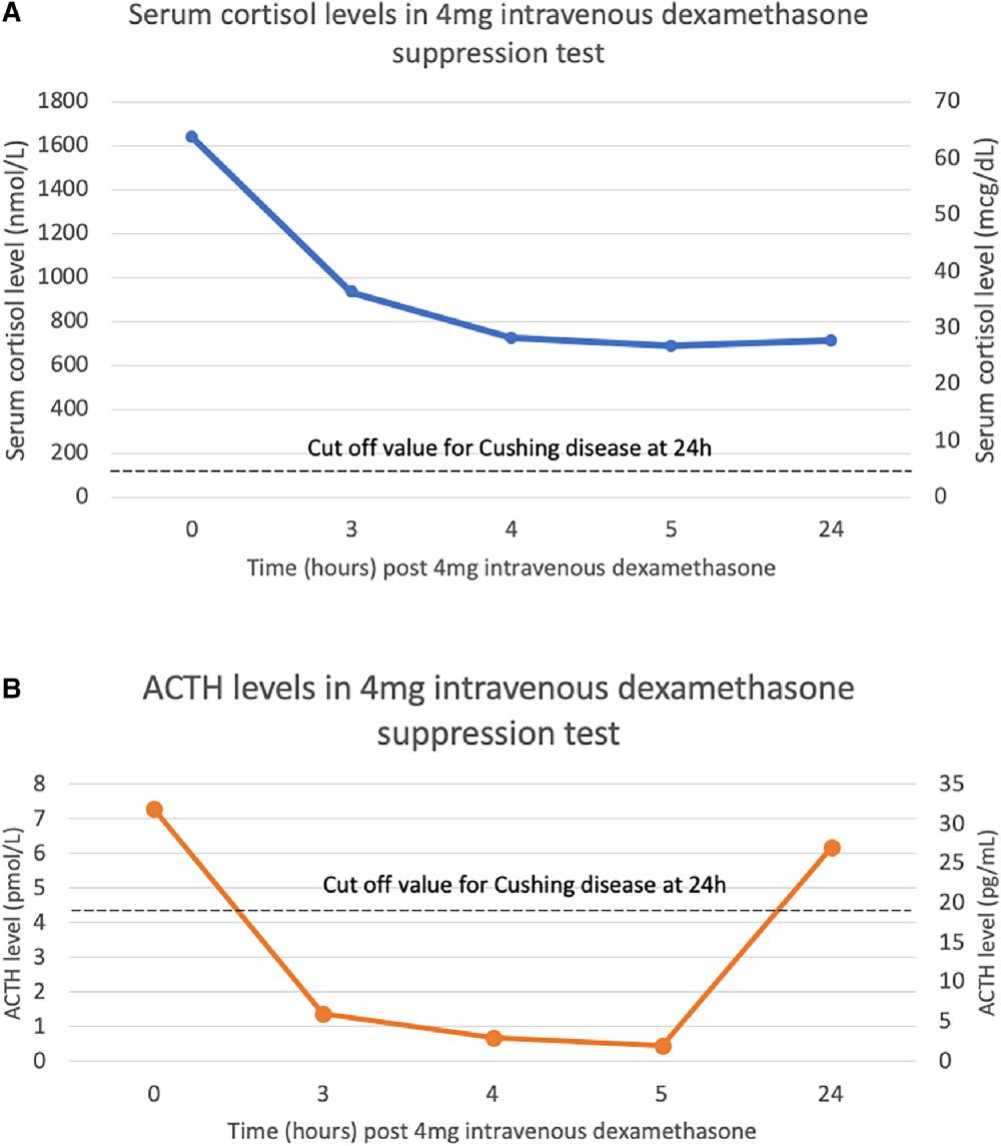
Results of 4-mg intravenous dexamethasone suppression test: A, cortisol levels, and B, adrenocorticotropin (ACTH) levels with suggested cutoff levels for Cushing disease [4].

Ketoconazole was commenced and titrated up to 400 mg 3 times daily, achieving 8 Am cortisol within the normal range, corresponding with improved confusion, affect, and weight gain. In preparation for a 4-mg intravenous dexamethasone suppression test, ketoconazole was withheld for 1 week, which resulted in subsequent elevation of cortisol burden with a repeat 24-hour urinary free cortisol (UFC) of 1377 nmol/24 hours (499 mcg/24 hours) (reference range, < 485 nmol/24 hours [<176 mcg/24 hours]), corresponding with clinical neuropsychiatric deterioration including increased confusion and agitation. Lumbar puncture did not reveal an infective, inflammatory, autoimmune, or paraneoplastic cause for the patient’s confusion and agitation. The 4-mg intravenous DST demonstrated significantly elevated cortisol and ACTH level at 24 hours (Fig. 2). Owing to the clinical deterioration with increased cortisol burden, ketoconazole was recommenced, with the aim to reassess ongoing need once nutritionally and medically optimized.

## Outcome and Follow-Up

After 1 month of nasogastric feeds, his appetite and oral intake had significantly improved and weight had increased to 51 kg (BMI 20 kg/m^2^), at which time ketoconazole was ceased. Three weeks after ketoconazole cessation, repeat 1-mg overnight DST was normal with 8 Am cortisol level of 26 nmol/L (1 mcg/dL) and 24-hour UFC was 334 nmol/24 hours (121 mcg/24 hours) (reference range, < 485 nmol/24 hours [<176 mcg/24 hours]). Psychiatry reassessment did not find evidence of any depressive, psychotic or paranoid features, or eating disorder. He remained well off ketoconazole without recurrence of neuropsychiatric symptoms off psychotropic medications and maintained normokalemia without supplementation. The initial significant weight loss was attributed to various gastrointestinal symptoms, fear of constipation due to hemorrhoids, and transient features of disordered eating. Overall, the diagnosis was PCS in the setting of malnutrition. Six months later, the patient remains well without cushingoid features with a mildly raised 24-hour UFC of 358 nmol/24 hours (130 mcg/24 hours) (reference range, < 280 nmol/24 hours [102 mcg/24 hours]), which is significantly lower than the initial 24-hour UFC of 2017 nmol/24 hours (730 mcg/24 hours) and 1377 nmol/24 hours (499 mcg/24 hours). The patient is to be followed for 6 to 12 months. His hypercortisolism is approaching remission; although cyclical CS with a long period is possible, we believe this is unlikely.

## Discussion

Differentiation between PCS and CD can be challenging because of their similar biochemical and overlapping clinical features including depression, obesity, and glucose intolerance. Dermatologic and musculoskeletal features such as easy bruising, skin thinning, and proximal muscle atrophy are more common in CD than in PCS [4]. While psychiatric conditions such as depression and eating disorders can cause PCS, neuropsychiatric and cognitive changes are also common symptoms of CS, reported in more than 50% of patients [5].

### Pathophysiology of Pseudo-Cushing Syndrome

Diverse abnormalities of the hypothalamic-pituitary-adrenal axis have been hypothesized to cause PCS [1]. In neuropsychiatric disorders, an exaggerated post-awakening surge in cortisol and lower activity levels of cortisol-deactivating enzymes 5-α-reductase and 11β-hydroxysteroid dehydrogenase type 2 have been reported [1]. In eating disorders, different mechanisms have been suggested including elevated corticotropin-releasing hormone (CRH) levels with normal ACTH, changes in the affinity of cortisol to corticosteroid-binding globulin, glucocorticoid action resistance, and reduced cortisol clearance [1].

### Differentiation between Pseudo-Cushing Syndrome and Cushing Disease

PCS and CD may present similarly biochemically, and may not be differentiated radiologically. CD patients may have normal or inconclusive MRI findings in 40% of cases, and pituitary adenomas may be seen in those with PCS [6]. Furthermore, BIPSS is unable to differentiate between the 2 conditions [2].

### Cortisol Burden

Although cortisol is elevated in PCS, the magnitude of hypercortisolemia is generally greater in CD. A retrospective study of 32 CD and 36 PCS patients found that 24-hour UFC was significantly lower in PCS compared to CD patients [4]. Similarly, a prospective study of 53 CD and 20 PCS patients demonstrated significantly greater cortisol levels in CD compared to PCS [3] (see Table 1). Interestingly, our patient with PCS demonstrated an atypically high cortisol burden, mimicking CD.

### Dynamic Tests

Dynamic tests can be used to attempt to differentiate PCS from CD. In the CRH test, peak ACTH and cortisol after injection of human CRH were lower in PCS compared to CD, with similar Δ%ACTH and Δ%cortisol [7]. In the 4-mg intravenous DST, CD can be differentiated from PCS by higher cortisol and ACTH levels at 8 Am on day 2 of the test [4]. However, in our patient with PCS, 8 Am cortisol and ACTH levels on day 2 were atypically elevated, meeting the threshold for CD (see Table 1).

In the desmopressin stimulation test, a rise in ACTH greater than 6 pmol/L (>27.2 pg/mL) is observed in CD (sensitivity 75-87%, specificity 90-91%), whereas PCS is less responsive to synthetic desmopressin due to differences in vasopressin receptor expression in ACTH-secreting adenomas [2].

The dynamic test with the most data and experience in PCS is the dexamethasone–CRH test. Dexamethasone is given orally (0.5 mg every 6 hours) for 2 days followed by intravenous 1 µg/kg human CRH 2 hours after the last dexamethasone dose. Serum cortisol and plasma ACTH levels are measured 15 minutes post-CRH. A CRH-stimulated cortisol level greater than 87 nmol/L (>3.2 mcg/dL) was found to be 94% sensitive and 100% specific for CD [3]; however various cutoffs have been proposed [8].

### Cortisol Circadian Rhythm

In contrast to CD, PCS retains the diurnal rhythmicity of cortisol secretion. A midnight:morning cortisol level ratio of more than 0.67 has been proposed to diagnose CD with 87% sensitivity and 0.67 or less to diagnose PCS with 100% specificity [3]. In our case of atypically high cortisol burden in PCS, midnight:morning serum cortisol level ratio (0.44) was the only investigation accurately predicting PCS and may warrant clinicians’ attention in such diagnostic dilemmas.

### Time as an Investigation Tool

In CD, signs and symptoms progress with time, whereas patients improve with resolution of the underlying cause in PCS. CD can however present as cyclical with periods of normal cortisol levels ranging from days to years [9], thus reassessment of the cortisol burden is prudent.

### Management of Pseudo-Cushing Syndrome

There is a paucity of literature focusing on the management of PCS. Management of the underlying condition often leads to resolution of hypercortisolemia. However, the role of adrenolytic therapies to suppress serum cortisol levels in PCS to prevent and ameliorate potential sequelae of elevated cortisol burden including immunosuppression and neuropsychiatric disturbance remains unclear.

In eating disorders, higher cortisol levels are observed compared to patients with depression, due to increased CRH. However, CRH can also independently act as an anorexic, further contributing to weight loss and leave the patients “locked in” [1]. Negative effects of functional cortisol excess have been described such as psychological and cognitive changes, attention deficit, bone loss, and oligomenorrhea [1]. Despite these potential sequelae, there are currently no formal recommendations regarding the treatment of hypercortisolism in such cases. In one case report, a 15-year-old girl with eating disorders and hypercortisolemia rapidly developed cushingoid features and hypokalemia post-weight gain, and subsequently was managed with bilateral adrenalectomy [10]. It is important to note that the role of reduction of hypercortisolemia in PCS and eating disorders has not been clearly elucidated. Moreover, the threshold of cortisol burden at which therapy may be beneficial and thus indicated remains unclear.

In conclusion, we present a case report of PCS secondary to malnutrition with an atypically high cortisol burden, complicated by immunosuppression and neuropsychiatric changes. The diagnosis was challenging because of the severity of hypercortisolism and dynamic tests in keeping with CD. However, phenotypically, the patient was not cushingoid, and the circadian cortisol rhythm was preserved. Treatment of hypercortisolism with ketoconazole corresponded with clinical improvement. PCS was confirmed as hypercortisolemia resolved after nutritional optimization. Further research is required regarding the role of cortisol lowering in PCS, especially in eating disorders.

## Learning Points

- Differentiation between CS and PCS can be challenging; however, it may be appreciated via its difference in dermatologic and musculoskeletal clinical features, the severity of the cortisol burden, dynamic test results, and progression with time.
- Assessment for the preservation of circadian rhythm may be helpful in patients with suspected PCS with an atypically high cortisol burden.
- Further research is required regarding the role of cortisol suppression/normalization in nonneoplastic hypercortisolism.

## Contributors

All authors made individual contributions to authorship. Y.P., F.G., I.S., and C.G. were involved in the diagnosis and management of this patient and manuscript submission. All authors reviewed and approved the final draft.

## Data Availability

Some or all data sets generated during and/or analyzed during the current study are not publicly available but are available from the corresponding author on reasonable request.

## Abbreviations

ACTH: adrenocorticotropin
BIPSS: bilateral inferior petrosal sinus sampling
BMI: body mass index
CD: Cushing disease
CRH: corticotropin-releasing hormone
CS: Cushing syndrome
MRI: magnetic resonance imaging
PCS: pseudo-Cushing syndrome
UFC: urinary free cortisol
